# "The Hospital" Nursing Mirror

**Published:** 1897-02-27

**Authors:** 


"The Hospital" Nursing Mirror.

Being the Nursing Section of "The Hospital."

[Contributions for this Section of "The Hospital" should be addressed to the Editor, The Hospital, 28 & 29, Southampton Street, Strand, London, W.C., and should have the word "Nursing" plainly written in left-hand top corner of the envelope.]

### News from the Nursing World.

### IRISH NURSES AND THE QUEEN'S COMMEMORATION.

A fund has been started in Dublin by a number of friends of the City of Dublin Nursing Institution for the purpose of making provision for nurses incapacitated from illness or by reason of age. A letter from Surgeon Wheeler in the *Irish Times* commends the scheme to the consideration of Dublin people, urging the desirability of a local commemoration of the Queen's reign. Surgeon Wheeler explains that the entire profits of the City of Dublin Nursing Institution, less 4 per cent. interest on the foundation capital are devoted to the purposes of the institution (which, though attached to the City of Dublin Hospital, is financially distinct from it), and that at the end of each year the largest available sum is divided as bonus amongst the nurses. Even then their earnings "are not at all sufficient to enable them to participate in the benefits of the Royal National Pension Fund, as their more highly remunerated sisters in England may do, where very much larger sums are paid by the public for nurses' services, and very much larger salaries can consequently be given." A committee has been appointed, upon which the Princess Edward of Saxe-Weimar, the Countess of Annesley, Viscountess Wolseley, and the Right Hon. Lord Justice Gibbon have, amongst others, consented to serve. Trustees have been appointed, and Mrs. Kildare Tracy has undertaken to act as hon. secretary.

### PRIVATE NURSING INSTITUTIONS.

We have warned nurses many times against putting their trust in nursing institutions "run" by private individuals, unless they are very sure indeed of what they are about. A recent case which has come to our notice makes us repeat that warning once more. A correspondent tells us of a nurse who, beguiled by "nice letters" from the head of a private nursing institution, joined it only to find that the place was notorious for overworking and under-feeding, that more than one nurse had broken down under the treatment, and she herself narrowly escaped a serious illness with which she was threatened, mainly in consequence of the inconsiderate treatment she had received. Nurses should make very careful inquiries before entering into agreements of this kind, and on no account bind themselves for any length of service without personal knowledge or some thoroughly reliable information.

### WORKHOUSE NURSING.

A letter in another column touches upon a very burning nursing question, one which, as we have pointed out frequently of late, will demand attention at head-quarters at no distant date. Everywhere boards of guardians are now finding the greatest difficulty in filling vacancies on the nursing staff of their infirmaries and workhouses. Advertisements are inserted time after time, and there are either no applications at all or they come from entirely unsuitable applicants. There are, of course, two potent reasons for this—the operation of the Superannuation Act, and, as we said last week, the growing difficulty in getting trained nurses to work under untrained matrons in the smaller workhouses. With regard to the first point, our correspondent suggests that guardians will best meet the present deadlock by stating clearly in their advertisements for nurses the total amount to be deducted in each case from the salary.

### COMMITTEES PLEASE COPY.

The committee of the Poplar Hospital for Accidents take a kindly and active interest in the recreations as well as in the work of their nurses. This year they have arranged to pay a subscription to Smith's Library for the benefit of the staff, so that a supply of new books shall provide amusement for off-duty hours. The idea is a most excellent one, giving great pleasure at small cost, and well worthy of imitation by the managers of other hospitals.

### A GENEROUS BEQUEST.

Mrs. Emmeline Fisher, of Abbotsbury, Newton Abbot, who some little time ago gave £5,000 to build a cottage hospital at Newton Abbot, in memory of her late husband, has just died, and has bequeathed a further sum of £2,000 for the furnishing of the new hospital.

### DISTRICT NURSING AT TWICKENHAM.

The Twickenham Nursing Association reports a greater number of patients cared for during the past year and an increase in annual subscriptions, with the satisfactory result that the association was able to close its year's accounts with a small balance in hand. Nurse Brasnett's ministrations, her "kindness, energy, and devotion to her duties," are warmly appreciated by the committee no less than by her patients.

### HOW PAUPERS ARE NURSED.

It is difficult to find words strong enough adequately to condemn the extraordinary action of the Tavistock Board of Guardians at a recent meeting. Some two months ago, it appears, a young, untrained girl had been appointed "assistant nurse" at the Tavistock Infirmary, and had since been reported by the medical officer as "far too young and inexperienced to be of use in such a capacity." This statement was borne out by him at a meeting held on the 19th inst., and corroborated by the head nurse (who is apparently herself uncertificated), two lady guardians, and several other members of the board, Mrs. Johnstone particularly speaking strongly on the subject, and drawing attention to the fact that no reliance could be placed on the "excellent weekly reports" from visiting guardians, for "the time to judge of hospitals and infirmaries and of the sufficiency of the staff was not at midday but at midnight, early in the morning, and late in the evening." In spite of all this an amendment was actually passed by 16 votes to

10 that the "assistant nurse's" services should be continued for six months, chiefly on the ground that she was "kind and sympathetic"! Here, therefore, at the Tavistock Infirmary, there are no less than 60 unfortunate sick people left to the care of one untrained nurse and a young girl stated by those who must know best to be quite unqualified for the duties put upon her. We are glad to note that the *Tavistock Gazette* of last week expresses a hope that the last has not been heard of this matter, and trusts that there are some humane people in the town who will take it up and not rest till this shameful state of things has been remedied.

### SECTARIANISM AT BOURNEMOUTH.

Last week we expressed a conviction that the committee of the Royal Victoria Hospital, Bournemouth, would speedily settle the question of the alleged "religious disability" when once it had been brought to their notice. At the annual meeting of the governors of the hospital last Friday the matter came up for discussion, and the chairman, Dr. J. Roberts Thomson, explained that no regulation existed or had existed requiring all probationers to be members of the Church of England, though an "unfortunate wording" in a type-written circular sent to intending candidates had conveyed that idea. Dr. Thomson added that instructions had been issued by the House Committee that in all future circulars the sentence in question should be erased.

### HULL SANATORIUM.

A very pleasant gathering of nurses and friends took place recently at the Hull Sanatorium to bid farewell to Miss Hardisty, the late night superintendent, previous to her departure for the Oxford Infectious Hospital, of which she was recently appointed matron. The chairman of the committee (Dr. Fraser) presented Miss Hardisty with a case containing silver sugar basin, cream jug, and serviette ring, from the nursing and domestic staffs, and expressed the regret felt by them at her departure.

### CHATHAM NURSING ASSOCIATION.

A branch of the Queen Victoria's Jubilee Institute for Nurses is to be established in Chatham this year, Mr. George and Mr. Thomas Winch having undertaken to contribute £50 per annum each for ten years if the town took the matter up and carried it through. It is proposed to start with two nurses, as promptly as they can be supplied by the institute, the annual cost being estimated at £160 to £200 for the two. Many Chatham people have come forward to help the scheme, which bids fair to be started on a thoroughly satisfactory basis. The medical men of Chatham have promised annual subscriptions of one guinea each.

### ON THE COAST OF LABRADOR.

Dr. Grenfell's letter to the February number of *Toilers of the Deep* is a most interesting one, with its graphic story of the work being carried on in Labrador by the Mission to Deep Sea Fishermen. In addition to the two hospitals at Battle and Indian Harbours, it is hoped soon to see a third hospital built at Harrington in Canadian Labrador. A Canadian friend has given £500 for the provision of a steam launch for the use of the doctor remaining at Battle Harbour during the winter, which will be a great help to these brave workers. Sister Williams has been at Battle Harbour Hospital for this winter, and Sister Carwardine is now in England. How badly medical aid and nursing is needed by the scattered population of Labrador may be guessed by one experience related by Dr. Aspland, who during last summer paid several visits of two or three days to one family, twelve in number, where eight children had scarlet fever, and three died. They lived in one small hut and outhouse, the nearest other house being across the water, three miles off. No one would come to help the poor things in their need, and for four weeks the father and mother nursed their children day and night. No wonder the visits of the Mission staff are welcomed with the deepest gratitude! Let those at home help by responding to the constant request for all kinds of warm clothing, boots—the look of gratitude with which these latter are received, says Dr. Willway, "makes one resolve never to wear one's own shoes beyond a certain time!"—blankets, and especially clothing for men and boys. The office of the Mission is at 181, Queen Victoria Street, E.C.

### BEDFORDSHIRE HOSPITAL-TRAINED NURSES' INSTITUTE.

We are indebted to Mrs. Rawson, the hon. lady superintendent of this institution, for an interesting report of the past year's work, the eighth year of its existence. The institute maintains private and district nurses, and a good step has been recently taken in forming a separate committee to superintend the latter branch of the work, and to keep the funds distinct from those of the private nursing branch. The committee have rearranged the working of the various districts with considerable advantage. Both funds were able at the close of the year to show a balance on the right side after payment of all outstanding liabilities, and this year it is proposed to give the private nurses a commission on their earnings. At the annual meeting very grateful thanks were tendered to Mrs. Rawson for her devoted work, a work which she has now carried on for nine years.

### SHORT ITEMS.

It is proposed that two district nurses shall be provided for Cannock and the surrounding districts, the cost of the scheme to be defrayed by contributions from the workpeople in the neighbourhood.—The Marchioness of Londonderry has become a member of the committee of the Queen's Commemoration Fund on behalf of the Jubilee Institute for Nurses. A sum of over £27,416 has now been collected for this fund.—Miss Kathleen M'Auliffe, Miss Kathleen Norcott, Miss Geraldine Mathews, and Miss Gertrude L'Estrange, have completed their training at the Red Cross Nursing Sisters' Home, Dublin.—The visiting nurse seems to be gaining a strong foothold in the United States, and a scheme of daily and hourly nursing has been adopted in many places with good success. — Three young Indian girls, one the daughter of a Pawnee chief, the other two belonging respectively to the Attawa and Wyandotte tribes, are stated to have trained as nurses in Philadelphia.—Sarah Bernhardt has consented to help at the grand fête in aid of the Victoria Hospital for Children this summer.—The Princess of Wales and Princess Victoria went to see the Norfolk and Norwich Hospital on their recent visit to King's Lynn.—On the 19th and 20th inst. two interesting performances of Pinero's "Sweet Lavender" were given at the Bijou Theatre, Archer Street, W. There was a large audience, and the proceeds of the two performances were about £40, which is to be handed over to the Children's Home Hospital, Barnet.—The Empress Frederick, who is staying with the Queen, is making a special point during her visit of going in a private capacity over several hospitals and other charitable institutions in London.—Last Friday an interesting discussion on "The Private Nurse" took place at the Trained Nurses' Club, Buckingham Street.

### Pharmacy and Dispensing for Nurses.

By C. J. S. Thompson.

IV. — DIURETICS — EMETICS — EMMENAGOGUES — EMOLLIENTS—EXPECTORANTS—HYPNOTICS — IRRITANTS — MYDRIATICS—NUTRITIVES—REFRIGERANTS—SEDATIVES.

Diuretics are medicines which promote the secretion of the urine. They include acid benzoic (useful in inflammation of the bladder), alcohol, solution of acetate of ammonium, ammonium benzoate and chloride, apocynium (given in dropsy as a diuretic), horseradish, borax, buchu (employed in complaints of the urinary organs, such as irritation of the bladder and urethra, &c.), caffeine, cantharides, caulophyllin, colchicum, convallaria, copaiba, cubebs, damiana, digitalis infusion, dulcamara, euonymin, hemidesmus root, iridin, juniper oil, lettuce, lithia water (effervescing), paraldehyde, tar, potass water (effervescing), pareira decoction, potassium acetate (used in dropsy), potassium nitrate, acid tartrate, tartrate, bicarbonate (useful in urinary affections where there is a deposit of uric acid), carbonate, chlorate, citrate and nitrate, solution of potash, senega infusion, broom, squill, simarubra, sodium bicarbonate, phosphate, sparteina, spirit of nitrous ether, turpentine, ulexine (used in cases of dropsy due to heart disease), elm decoction, and bearberry leaves.

Emetics are medicines which excite vomiting. The following are chiefly used: Alum in repeated doses of a teaspoonful in honey or treacle, chamomile (infusion in large doses produces vomiting), tartarated antimony as an emetic from one to two grains, apomorphine hydrochlorate (a powerful and prompt emetic, usual dose one-tenth grain injected hypodermically), baptisin, copper sulphate (five to ten grains will induce vomiting), ipecacuanha powder (as an emetic, fifteen to thirty grains), mustard (in powder—a tablepoonful in half a pint of water), sodium chloride (common salt)—in large doses in water; (in case of a leech being swallowed, a strong solution of common salt should be given), tobacco, zinc acetate, zinc sulphate (as an emetic from ten to thirty grains).

Emmenagogues are medicines which maintain or restore a healthy condition of the menstrual discharge and increase the quantity. They include compound decoction of aloes, pill of aloes and myrrh, ammonium chloride, apiol, borax, calendula, cimicifuga, ergot, reduced iron, black hellebore, manganese oxide, potassium permanganate (useful in amenorrhœa), quinine, rue, savin, and senega.

Emollients are substances which soften and relax the tissues, and are useful in protecting sensitive surfaces and allaying irritation. They are best used in combination. They include lead, lanoline, almond oil, white wax, spermaceti, collodion, glycerine, glycerine of starch, olive oil, prepared suet, vaseline, &c.

Expectorants are medicines which promote the secretion of bronchial mucus. The following are chiefly used: acid benzoic, ammonia, ammonium carbonate (useful in pneumonia and bronchitis), ammonium benzoate and chloride (in chronic bronchitis internally, and by inhalation), ammoniacum (employed in chronic catarrh and asthma), oil of aniseed, tartarated antimony (given in continued small doses to relax the mucous membranes and skin), apomorphine hydrochlorate (used in croup, bronchitis, and bronchial catarrh), asafœtida, balsam of Peru, and balsam of tolu (largely given in chronic catarrh, asthma, and pectoral complaints), benzoin (employed in form of compound tincture for chronic cough, internally and by inhalation), copaiba, cubeba, codeine, ether, galbanum (useful in chronic affections of the mucous membrane), ipecacuanha (valuable in bronchial complaints), larch, lobelia, myrrh, tar (in chronic bronchitis, internally or by inhalation), quillaia, squills (a popular remedy in the form of syrup; increases the secretion of the bronchial mucous membrane, and aids expectoration of mucus in chest complaints), senega (chiefly used in chronic bronchitis), styrax, carbolic acid, chlorine, creasote, and iodine (by inhalation in bronchitis, and chronic congestion of the larynx).

Hypnotics or soporifics are medicines which induce sleep. They include amyl nitrate, boldo, butyl chloral hydrate, monobromated camphor (given in hysteria and delirium tremens), Indian hemp, chloral hydrate, chloralamid, chloroform, codeine, conium, creasote, henbane, hyoscyamine sulphate, hyoscine (used hypodermically), hypnone, hops, methylal, morphine and its salts, narceine, opium, paraldehyde, piscidia, potassium bromide, sodium bromide, sulphonal, somnal, urethane, stramonium, chloralose, papaverine, bromal, acetophenone, amylene hydras, bromidia, chlorobrom, hypnal, metaldehyde, tetronal, trional, uralium, and scopolamine hydrobromate

Irritants are substances which stimulate and cause irritation or inflammation of the parts to which they are applied; they differ in their intensity of action, and may be divided as follows:—Rubefacients when applied to the skin produce local warmth and redness. They include alcohol, ether, liquid ammonia, warming plaster, pitch plaster, compound camphor liniment, capsicum liniment, chloroform liniment, iodine liniment, compound mustard liniment, mezereum, cajepute oil, lemon oil, rosemary oil, and also the oils of rue, amber, and turpentine. Vesicants are agents which raise a vesicle or blister, as glacial acetic acid, strong liquid ammonia, and cantharides. Pustulants are those which produce a pustule, such as tartarated antimony, nitrate of silver, and croton oil.

Mydriatics are agents which produce dilatation of the pupil of the eye. They include atropine sulphate, belladonna, daturine, duboisine sulphate, homatropine, hydrobromate (more rapid in action than atropine), hyoscyamine, and scopolamine.

Nutritives are substances which quicken assimilation and improve the condition of the living tissues. They include gum acacia, sweet almonds, malt extracts, meat extracts, Irish moss (used as an article of food on the west coast of Ireland, where it is found), Iceland moss decoction, figs, glycerine, milk, manna, arrowroot, cod liver oil, olive oil, yolk of egg, prunes, sugar of milk, suet, mixture of brandy, treacle, and raisins.

Refrigerants are medicines which diminish the body heat and quench thirst. The following are chiefly employed: Acetic acid diluted, citric acid, hydrochloric acid diluted, nitric acid diluted, phosphoric acid diluted, tartaric acid, sulphuric acid diluted, solution of ammonium acetate, orange juice, lemon juice, solution of citrate of magnesia, mulberry syrup, lemon syrup, oxymel, citrate of potassium, chlorate of potassium, nitrate of potassium, acid tartrate of potassium, prunes, spirit of nitrous ether, tamarinds, and pure water. A weak infusion of cascarilla bark is recommended to quench thirst during fevers.

Sedatives are agents which exert a soothing influence on the system by diminishing pain, lessening functional activity, or tranquilising disordered muscular movement. They may be classed as follows:—Local Sedatives include acid carbolic (given as a sedative to check sickness and flatulence), acid hydrocyanic (used to allay vomiting), atropine, belladonna, borax, chloral hydrate, creasote, morphine, opium, acetate of lead, and solution of subacetate of lead (externally for inflammation arising from sprains and bruises). Pulmonary Sedatives: Acid hydrocyanic, bromide of ammonium, belladonna, oxalate of cerium, gelsemium, cherry laurel water, lobelia, morphine, opium, wild cherry bark (useful in the form of syrup for spasmodic cough), and stramonium (employed also in convulsive coughs). Spinal Sedatives: Ammonium bromide, monobrated camphor, gelsemium, bromide of nickel, Calabar bean, potassium nitrate and bromide, sodium bromide, green hellebore (in spinal spasms), viburnum (as a sedative to the uterine nervous system), and bromide of zinc. Stomachic Sedatives: Arsenious acid, carbolic acid, hydrocyanic acid, phosphoric acid diluted, nitrate of silver, belladonna, Bismuth salts, solution of zinc, oxalate of cerium, chloroform, cocaine hydrochlorate, creasote, mercury with chalk, henbane, opium, potassium bicarbonate, effervescing, potass water, sodium bicarbonate, effervescing soda water, sodium bromide, and zinc oxide. Vascular Sedatives: Aconite, nitrate of amyl, tartarated antimony, apocynum, cherry laurel water, colchicum, digitalis, ergot, ipecacuanha, nitro-glycerine, acetate of lead, potassium nitrate, sodium nitrate, spirit of nitrous ether, and green hellebore.

### How Nurses Can Commemorate the Year 1897.

By HELEN FOGGO THOMSON, Secretary of the Victoria (Commemoration) Club.

IN February, 1890, I read a paper before the Royal British Nurses' Association on private nursing, in which I embodied the following:—

"We all know the nursing profession is still in its infancy, and it is only of recent years that special training has been seriously expected in a private nurse. Formerly a certain amount of empirical experience was all that was looked for, and this was too often associated with a roughness of manner and deportment which would not have been tolerated in a domestic servant. In this we have made rapid strides, and though even now the training and skill of the private nurse may often leave much to be desired, I do not think any profession can show a greater advance in the personal qualities of its members than is seen by comparing the average nurse of to-day with her predecessor of the last generation."

So marked has this improvement now become that there is a real danger lest some should flatter themselves that they have reached perfection. Those, however, who know most of the subject are keenly aware that there is still abundant need for improving the training, knowledge, and efficiency of nurses, as a body, when they have left the hospital. Our advance as a profession has not kept pace with our progress as individuals. Indeed, the former has been little enough, but we have made a beginning. We have our journals, which can speak in the name of the nurses as a body, but it is to our united action that we must look for the improvement of the general condition of nurses; for increasing their professional usefulness, for securing them the due reward of their labour, for ameliorating the conditions under which they work, for redressing the grievances from which they undoubtedly suffer, and it is to these matters I would now especially invite attention.

As one who has been a private nurse, and knows and has worked with many others, I would say that the great drawback and disadvantage under which we labour is that we tend to become rusty, to fall into a narrow groove, to forget our clinical experiences, and, above all, to have no opportunity of learning, of hearing of, or seeing improved methods of nursing, nursing appliances, new dressings, and so forth. This at first is felt keenly, then one becomes reconciled to it as inevitable, and so almost imperceptibly one glides into the vegetation stage.

In connection with this I would like to suggest a scheme which has for some years commended itself strongly to my mind, and which I have had no opportunity until now to bring to fruitful issue, viz., to organise post-graduate lectures for nurses upon all subjects connected directly or indirectly with their work, and exhibitions of the most recent nursing inventions, appliances, and dressings. I cannot but think that such an exhibition would not only be interesting, but of considerable educational value as well, and I think the time has now come for nurses (like any other national body of workers) to amalgamate and decide what they want; and then with the courage of their opinions, and the necessary *esprit de corps*, to obtain it.

The Victoria Commemoration Club has been organised at considerable expense to fulfil this great need, and I do most sincerely hope that nurses will appreciate the spirit in which it has been started, and come forward and support it as they ought. There never will be a better opportunity afforded them of combining on neutral ground. Of late years there has been too much disunion and party spirit shown in the nursing profession, and so many opportunities given for the outside world to criticise us unfavourably. This must have been most distasteful to the better-class nurses. No doubt, now that we have originated the idea, many mushrooms will spring up, and small nurses' clubs all over London will be the consequence, but I think (knowing the profession as I do) that nurses will not be slow to realise that to improve their social status as a body, and to keep in touch with the progress of the times, they must co-operate and unite with the main body.

### Appointments.

### MATRONS.

RADCLIFFE INFIRMARY, OXFORD.—From amongst a large number of candidates Miss Agnes J. Watt has been chosen to fill the vacant post of Matron at this hospital. Miss Watt was trained at the London Hospital, where she has since held appointments as sister of accident and surgical wards, and won for herself excellent testimonials. She will be greatly missed by many friends at the London, and takes with her cordial good wishes for her future success. The recent history of the Radcliffe Infirmary goes to show that the newly-appointed matron will have a difficult task before her in bringing her department up to modern standards, matters having now become so unsatisfactory that Lord Dillon and the Rev. C. Fletcher have both been compelled to resign their connection with the institution. We sincerely trust that, having secured a good matron, the management will now support her in the efficient administration of the nursing department.

CITY ISOLATION HOSPITAL, NORWICH.—Miss Mary Gardner has been unanimously selected to fill the post of Matron at this institution. Miss Gardner trained at Crumpsall Infirmary, Manchester, and the Mildmay Hospital, London, subsequently holding appointments as staff nurse at the Birmingham General Hospital, night superintendent at the Cardiff Infirmary, matron of the Llandudno Convalescent Home for Women, and, latterly, as night superintendent and deputy-matron at the Nottingham Borough Isolation Hospital. Her testimonials are excellent.

THE WORKHOUSE INFIRMARY, BRADFORD.—Miss J. A. Smith has been appointed Superintendent of this institution. Miss Smith was trained at the Birmingham Workhouse Infirmary, where she has held the post of sister-in-charge for three years.

HERTFORD AND WARE JOINT HOSPITAL.—Miss Isabella McIntyre has been appointed Matron to this hospital for infectious diseases. Miss McIntyre was for over four years matron of the International Hospital, Naples.

### Seven Weeks in a London Fever Hospital.

By a late Patient.

II.

And now I have gone pretty well through the routine, and the days become monotonous. My only resource is reading, and though I have morning and evening papers sent in, there seems a dearth of interesting news, and time hangs heavily. Of conversation there is little. The beds are too far apart to encourage it between patients, and the nurses, though patient and courteous, are usually too much absorbed in their duties for mere idle talk.

Most of the cases in the ward seem slight. Opposite me, however, are two young men whose cases are complicated by cold. The slightest chill after scarlet fever leads to kidney troubles, dropsy, &c., and this is why the authorities are so careful not to let patients leave their beds a day too soon.

In the bed next me is a lad of eight. He has a bad attack, including a severe sore throat. Poor child ! he is very thin and worn—the children suffer most in these cases. At times he is delirious. The great difficulty is to make him swallow his milk—the only nourishment he can take. He sleeps badly, and as I, accustomed to late hours, do not go to sleep at nine o'clock, I often have occasion to note the unwearied vigilance of the night nurses. Half a dozen times in the early night some sound of suffering breaks from the child—a cough —a groan—some incoherent babbling—or he tosses uneasily. However slight the sound or movement, it has hardly caught my attention when the silent figure of the night nurse glides to the bedside—to bend over it—to help the patient to a drink—to smooth the pillow, or change the ice-bag on the head. Nothing could be more admirable than the watchfulness of these women. And now time creeps slowly. Sometimes the tedium is beguiled by a game of cards. Three of the patients who are allowed to get up, and are arrayed in the blue hospital suits, seat themselves at the bedside of a fourth, and a couple of hours pass pleasantly enough at whist. Then when the sick boy of whom I have written is sufficiently recovered to make merriment harmless, a concert is improvised. A small harmonium is requisitioned, an accompanist and singers found, and the ward soon echoes to the dulcet strains of "I'm getting ready for my mother-in-law," "It's a great big shame, and if she belonged to me," and kindred music-hall ditties. We have a strong force present in the person of a "theatrical" who has in his time "played many parts," from Hamlet in the travelling company to clown in a circus, and is at present a trainer of performing animals. His little son is in the hospital, but in the convalescent ward, and his father brings him in to perform acrobatic feats wonderful in so small a child. Then the father, with an assistant, gives us a "humorous sketch," which is much applauded, and the evening closes pleasantly enough. This is a farewell entertainment, for next day the actor and half a dozen others are going to Winchmore Hill, the convalescent hospital in connection with this, to finish their "cure."

The next few days are dull, but I discover that one of the nurses has been to Jersey, and we compare notes of that charming island. I also discuss the joys (in anticipation) of "Bonnie Scotland," with a "braw Scotch lassie" who is in charge of our ward during the day. She waxes eloquent on the topic of her beloved country, and loves even the bag-pipes, but admits they sound best at a distance. I suggest that a hundred miles is the best distance, but she won't allow this. And now it is eight days since my arrival, and I am carried to the convalescent ward and put to bed there. It seemed very little different to the one I had left, save that most of the patients are up; some all day, others for an hour or two each day. Next day I am seen by the doctor, who announces, to my great delight, that I may get up for a hour or two each day. The nurse in charge routs up a blue serge suit, which, if not quite a Bond Street fit, "will serve." A coloured cotton shirt (I already have a flannel undershirt), a pair of woollen stockings, and lace-up boots complete the attire in which I can walk about the ward.

Next day is Sunday. I spend it in the same way; not being able to leave the ward, I cannot attend the service which the chaplain conducts in an adjoining building. And now I feel quite well, save for slight weakness. The "peeling" process is fairly advanced, the skin having fallen off my chest and hands. This is assisted by the bath, taken every other night. Then follows permission to go out in the grounds, and I am able to take stock of the outer aspect of the hospital.

There are eighteen long wooden huts standing in three somewhat irregular rows, divided from each other at the sides by thirty feet of grass plot and at the ends by the wall-less corridors to which I have alluded. The doctor's house, surrounded by a pleasant garden, stands in a corner of the grounds. There is an engine-house, whence the steam is obtained for the hot water pipes which warm the wards, and a coal shed. A broad gravel path runs right round the whole group of buildings, and outside it lies a field which is being converted into a recreation ground. We can roam over the grounds (except a small portion reserved for female patients), but a rigid discipline prevails. We must not speak to the female patients, or even to the nurses, as you render the latter liable to suspension or dismissal if they hold conversation with you.

I soon got tired of the routine again, and asked and obtained the doctor's permission to be taken to Winchmore Hill. So on the morning of June 11th, a fortnight after my arrival, I am taken, in company with other men and several children, to that convalescent hospital, being accompanied in the omnibus by a nurse who takes the papers describing our cases. On entering the gates of our new abode we pass through a considerable amount of ground laid out in flower beds and gravel walks; but not, alas ! for the patients' use. We perceive a number of brick buildings standing a little distance from each other. These are the wards or "pavilions," of which there are eighteen. Each has a piece of ground like a school play ground attached to it, with swings, &c.; and also a good-sized field for cricket, &c.

I learnt that the hospital was able to accommodate about twelve hundred patients. At present there were about seven hundred who came from the various fever hospitals under the control of the Metropolitan Asylums Board. On our arrival we entered one of the brick buildings, and proceeding to the bath-room, divest ourselves of our clothes (which go back to the hospital whence we came), and receive in exchange a similar suit, in which, however, the trousers are of white corduroy. We wait an hour and a half till the doctor comes and duly passes us as convalescents. He asks if we want the allowance of beer and tobacco, and receives an affirmative reply. This consists of a pint of ale or porter per day and a quarter of an ounce of shag tobacco.

We now march off to the dining-room. The meal is of roast beef, with potatos, greens, and bread, followed by rice pudding. For drink either the ale or porter or lemonade. I was much disappointed in the fare. The food was much coarser than in the North-Eastern Hospital, and the porter or ale possessed nothing in common with anything I had ever consumed under these names except the colour. We were afterwards shown our bed-rooms. It held fourteen beds, and as it served for sitting-room as well, was furnished with a deal table, forms, and a bagatelle board. After dinner we went into the field, and I joined in a game of cricket, which promised to be my only recreation, save a morning paper of the charge nurse's, which was given to us in the afternoon. I had heard that at convalescent hospitals there were usually libraries, or at least reading-rooms with books, but here was there neither reading-room, smoking-room, nor sitting-room, except the one which served all these purposes in addition to that of sleeping. Nevertheless one soon dropped into the daily routine. Breakfast at six o'clock, tea, bread and butter, and egg. The field not being open till after nine we then went into the yard (after making our beds). Then perhaps cricket. Luncheon at ten o'clock of bread and cheese, milk or lemonade. Then more cricket till dinner (about one.) This consisted, with painful monotony, of very tough and coarse beef, potatos and greens, with the addition of the doubtful liquor called beer. Tea at four o'clock—bread and butter or marmalade. At seven-thirty, supper—the same as lunch. The cricket field closed at eight o'clock, and we then came into our room, to smoke or play cards till bed time (nine o'clock).

A complaint made to the doctor of the diet produced some small improvement. The meat was a little better, and the beer (formerly fetched in jugs and allowed to stand for three hours before dinner) was brought in stone jars. Of course in a large place of this character the patients cannot expect everything of a high quality, but I maintain that some great improvement could be introduced with little or no additional expense. The lack of variety in the diet might be remedied cheaply enough. The beer might surely be kept in the barrel. The provision of a reading room and a few papers and books need not be ruinous.

I was much struck by the fact that the pleasant garden grounds which I have mentioned were a sealed book to us, being reserved for the staff. I am far from saying that the staff should not be comfortably provided for in all respects. But surely the *raison d'être* of an institution like this is the benefit of the patients. However, I managed to get through five weeks without, I suppose, more than the inevitable feeling of weariness, until at last, the "peeling" of my feet being complete, I was told by the nurse that I might "show up" to the doctor. So one morning I found myself one of a row of patients who, sitting on a form in our bed-room, submitted their feet to that official's examination. Mine were found in a satisfactory condition, so I was "passed," and after a delay of two days occupied by the superintendent in communicating with my friends, and arranging for my clothes to be sent to the discharging hospital, I am driven there in an ambulance, and, after a bath, in which carbolic was an ingredient, allowed to don my own clothes, and go forth into the outer world after a period of seven weeks under the protecting care of the Metropolitan Asylums Board.

H. S. N.

### Our American Letter.

(COMMUNICATED.)

CONSIDERABLE alarm was caused in January by the sudden discovery of flames bursting from the Bellevue Hospital Medical College, in East Twenty-sixth Street, New York. The building was seriously damaged before the fire could be arrested, the upper part being completely burned out. Although so close to the hospital that the flames could be seen in several of the wards, there was no alarm among the patients.

The twelfth anniversary of the New York Post Graduate Hospital took place in January, and was duly celebrated by a pleasant entertainment at the hospital. After some music Dr. Emmet, the treasurer, read a statement respecting the work of the hospital, and announced that the coming spring would see the completion of a training school for nurses, such as would provide well for the needs of the patients.

The plans for the new and magnificent hospital which Mr. J. Pierpont Morgan has offered to give to the Society of the New York Lying-in Hospital have been prepared by Mr. Robert H. Robertson under the supervision of Dr. James W. Markoe, who has made a special study of similar institutions abroad. It proposes to be a 10-storey brick and stone building, with accommodation for 250 beds and capacity for working, in conjunction with the Sloane Maternity and the Bellevue Emergency, the whole outdoor obstetric work of the City.

The first annual meeting of the Associated Alumnæ of the Brooklyn Training Schools for Nurses was recently held in the Hoagland laboratory, over which Miss Merritt, Superintendent of the Brooklyn Training School, presided. A vote of thanks was passed to Miss Merritt, the retiring President, for her untiring efforts on behalf of the association, of which she was the chief originator. The association has only been organised for just a year, and has every reason to be satisfied with the success of the registry which it has founded. Now committees are to be appointed to arrange other branches of the educational and social work.

There has been recently an unaccountable outbreak of typhoid fever amongst the nurses of the Mount Sinai Hospital, some twelve cases in all. All the nurses are fairly on the road to recovery now, and there have been no fresh cases. The cause is a mystery. None of the nurses had been in contact with the disease, and the drainage of the institution was not believed to be in fault.

The Training School for Nurses in connection with the Long Island College Hospital, of which Miss Sutliffe is lady superintendent, is in future to give its probationers a three years' course of training. The announcement of this proposed change was received with much satisfaction by the members of the Alumnæ Association at their last quarterly meeting.

The recent action of the Secretary of the Treasury regarding Canadian nurses has given rise to much discussion and anxiety in the hospital world in the States. The immigrant inspector at West Superior, Wis., has been advised that St. Luke's Hospital, Duluth, cannot accept Canadian probationers if it pays them for their services. The immigrant inspectors all along the border are to secure lists of all Canadian nurses in the institutions under their control, and while no present probationers are to be deported, the lists are to serve to check any future arrivals. The injustice of the new law is that it applies to nurses only, not to other hospital attendants. It is not to be retrospective, and does not apply to Canadian nurses who have already graduated. The *Trained Nurse* says on the subject: "The question is a serious one to American hospitals. Practically there are more Canadian nurses in the country than American to-day, two to one, and this is so because there is so little professional work in Canada for the better class of women, and nursing as a profession is looked upon there as in England, because in the mother country royalty has set its seal upon it. Whether the ruling of the Secretary be popular or not, whether it is equitable in the sense that it rules against nurses while admitting other hospital attendants, it certainly will do no injustice to those Canadian friends of ours who have made their home and received their professional training here, while it will open the way to the great body of American womanhood to enter the breach of this ever-widening profession."

### Everybody's Opinion.

[Correspondence on all subjects is invited, but we cannot in any way be responsible for the opinions expressed by our correspondents. No communication can be entertained if the name and address of the correspondent is not given, or unless one side of the paper only is written on.]

### WORKHOUSE NURSING.

"Interested" writes: Your paragraph in the Nursing Mirror, February 20th, headed "Nursing at the Bow Infirmary," gives material for somewhat anxious consideration. I gather from it that the vacancies on the nursing staff are filled up by nurses from Guy's Hospital and the Nurses' Co-operation, and although the word "temporary" is not used, I conclude that such appointments cannot be permanent. This being the case, it appears a far from satisfactory state of things that the Board of Guardians of a poor union should be obliged to fill up nursing vacancies at a cost of not less than one or two guineas a week, and this at a time when no epidemic, such as influenza, is raging. It is not probable that the institutions mentioned have made reductions from their usual charges (there is no reason why they should do so), and the present cost roughly estimated for one week is slightly more than an assistant nurse's salary for the whole year. This heavy expense is caused by no fault of the Board of Guardians, but results to some extent, I conclude, from the extreme difficulty experienced just now in getting good trained nurses to apply for Poor Law appointments. I should be glad if through your columns the experience of matrons and other experts could be obtained as to the reason of this difficulty. It may, in my opinion, largely be due to the passing of the Superannuation Act, and to the wording of Poor Law advertisements, in which percentage deductions are almost invariably stated to be a condition of appointment, but in which the *amount* of these deductions is seldom named. Nurses will have, we hope, soon power to contract out through the Amendment Bill. But while the present Act is in force it would be a more business-like plan for all Poor Law advertisements to contain not only the salary offered, but the exact amount which will be deducted both from it and from all emoluments. Nurses are unlikely to become candidates for appointments while such necessary information is not clearly stated. Poor Law salaries are none too high at present, and a reference in the advertisements to deductions is scarcely likely to act as an added attraction to would-be candidates.

### MEDICO-PSYCHOLOGICAL ASSOCIATION AND ITS NURSES.

Miss Jessie B. Bisset, Wilts County Asylum, writes: I have read with interest the correspondence relative to the proposed admission of asylum-trained nurses to the register of the Royal British Nurses' Association as mental nurses. This question has called forth a good deal of feeling. The proposal has been described as "at once senseless and absurd," as "one mass of injustice and error," and of infringement and fraud. The promoters have been treated with pharisaical lectures; hospital-nurses have been called upon to resist the introduction of living lies into the Association, and Miss Wingfield has solemnly stated that she has no doubt that if this deplorable measure is thrust upon the hospital nurses, the best class of them will remove their names from the register. Altogether hospital nurses are very much horrified at the degradation which they imagine they are to be subjected to, and I think it might be as well to remind them that asylum nurses of to-day are not the old lunatic keepers (as one nurse calls them) of the past. In my opinion the Association cannot be degraded by adding to its sphere of usefulness, nor can the public be defrauded by the proposed registration of trained asylum nurses (under a special heading) who have passed through a rigorous curriculum of training, practical as well as theoretical, and an examination in no way inferior to that of the hospital nurse. Tradition and popular ignorance associate the asylum nurse with all that is rough and uncouth, but a day spent in one of our asylums would quickly dispel so erroneous an idea. Hospital nurses without asylum training cannot undertake the nursing of mental cases, for they have had little or no opportunity of seeing the various forms of insanity, and have no knowledge of these difficult and often dangerous cases, which must be treated and nursed as well as any other form of disease, and which require, on the part of the nurse engaged, special training and experience. In my opinion no nurse is fully qualified unless she has passed through all the branches of nursing, viz., medical, surgical, fever, midwifery, and mental; and therefore I cannot see why the register of the Royal British Nurses' Association should be exclusively kept for those who have only had medical and surgical training.

### THE JOHANNESBURG NURSES.

Nurse "C. E. J." writes: In reference to what has lately been said regarding the Johannesburg Hospital, I should like to state (as I am one of the thirty who went out, and, I am sorry to say, had to return after an attack of typhoid fever) that I think it very unfair to warn nurses against going to the Johannesburg Hospital, where good nursing is so much needed, and especially after all the kindness and courtesy we received from everyone there. As we went out to reorganise the nursing, we could not of course expect to find everything perfect, and as regards the two meals a day, the food is fairly good, and we certainly had breakfast, lunch, dinner, tea, and supper. The work is very hard, and more trying than in England, especially at first, but everything that was asked for we got in time. The nurses' quarters were rather cramped, so the board promised to build more rooms, which they are about to start. I hear that they have a pianoforte and an organ, and the hospital and nurses' home is to be lighted with electric light. It is only in accordance with the terms of our agreement that nurses leaving the hospital before their time was up should forfeit £100.

*** It is always well to hear both sides of a disputed matter, and we very gladly publish the above letter, which puts a different construction upon the action of the nurses who recently broke their contract and left the Johannesburg Hospital. We have seen our correspondent and heard her views on the whole matter. She was herself invalided home after typhoid fever, and is anxiously awaiting the first opportunity of going out again with the view of rejoining the hospital staff. She tells us that with regard to the complaints made as to food and accommodation, the former was sufficient in quantity, and in quality "better than is provided in some English hospitals"; and the latter, though somewhat cramped in space, each nurse being allotted a small cubicle, not nearly so uncomfortable as represented. So far as the pay was concerned, the nurses having their entire uniform provided, their passage paid, and no expenses in the way of board, received in salary a clear £55 per annum. The high price of living in Johannesberg, which raises ordinary wages in a corresponding degree, would not, therefore, affect the salaries of the nurses, who could not at this rate consider themselves as otherwise than adequately paid. Nurse "C. E. J." tells us that the board were in all ways considerate and kind to the staff and ready to meet their wishes, the gradual reorganisation and improvement of the hospital being but a matter of time. The action of the nurses has made matters very much harder for the rest of the staff.—Ed. *T. H.*

### NURSE OR HOUSEMAID.

A Private Nurse writes: Allow me to endorse most emphatically all that "A Staff Nurse" says in your issue of the Nursing Mirror of January 30th, 1897, with regard to more "domestic" aid being given in the wards of our hospitals at home. When I entered the lists as a probationer I was far beyond the usual age for such, and my motive for doing so was to learn "scientific nursing," not to clean locker tops and polish brass fittings. Had I wished for this latter accomplishment, I could have ranged myself as a "charwoman" years before. My objections to this "char" work (let me call it) were not from any silly, childish dislike to what your correspondent "Staff Nurse" calls menial labour, but were sensible and substantial reasons. First, personally I had no time to waste, being, as I have already stated, past the usual age of probationers, in doing work I knew already better than the generality of educated women. Second, a woman who does all sorts of cleaning and polishing cannot possibly keep her hands soft, tender, and cool, and such as a sick nurse's ought to be, and such as sick people love to hold and feel. In conclusion, let me say that long years before a hospital ward ever saw me, I had had experience in all sorts of sick nursing, both on land and sea, at home and abroad; but later, when circumstances allowed me and the opportunity offered, I desired to "move with the times," and have some hospital training, and having received this latter, I am now private nursing in South Carolina, U.S.A.

LADY PRIESTLEY AND THE NURSES.

"J. W." writes: As a trained nurse of several years' experience I should like to say a few words in regard to the various letters commenting on and condemning both Lady Priestley's and Mr. Hall Caine's recent attack on nurses. There is no doubt the life of a nurse is both hard and wearing, and she requires recreation perhaps more than other people; also that the "labourer ought to be worthy of his hire"; but as her faults and virtues have been conspicuously shown forth, would it not be well for us nurses (as well as those who employ us) to bear this fundamental law in our hearts that "Charity endureth all things" as well as "thinketh no evil," and that kind words and deeds help far more than bitter retaliation?

### Minor Appointments.

DERBY COUNTY ASYLUM.—Miss Harriet Kendrick has been appointed Chief Nurse at this asylum. She received her training at the City of London Asylum, Stone, Dartford, subsequently becoming head attendant at Chartham Asylum, Kent.

POPLAR AND STEPNEY SICK ASYLUM.—Miss Kathleen Burleigh has been appointed Assistant Matron at this infirmary. Miss Burleigh was trained at St. Bartholomew's Hospital, where she has since held the position of staff nurse.

METROPOLITAN ASYLUMS BOARD TRANSPORT DEPARTMENT (SMALL POX).—Miss Jane Edwards has been appointed Nurse Superintendent of this department. She received her training at the South Devon and East Cornwall Hospital, Plymouth, subsequently holding appointments as charge-nurse at the Borough Fever Hospital, Plymouth, and the Isolation Hospital, Faversham, Kent.

TIMPERLEY, CHESHIRE.—Sister Ruth Cumberland has been appointed District Nurse at Timperley. Sister Cumberland, who was trained at King's College Hospital, and holds the L.O.S. diploma, has had large and varied experience in district, hospital, and private work. Her last appointments were as district nurse at Hulme, Manchester, and district nurse and midwife at Hampton Wick. During seven years' excellent work at the latter place she earned for herself universal regard and esteem. She holds very good testimonials.

### Where to Go.

EAST LONDON NURSING SOCIETY.—The annual meeting of the East London Nursing Society will be held at the Mansion House, by permission of the Lord Mayor, on Monday, March 1st, at three o'clock.

THE VICTORIA (COMMEMORATION) CLUB, THE HOSPITAL BUILDING, 29, SOUTHAMPTON STREET.—A lecture on the "Chemistry of Food" will be given at this club on Tuesday, March 2nd, at 3 p.m., by Mrs. Alting-Mees (gold medallist on hygiene, &c.). The lecture will deal with the laws of chemistry of food, briefly touching on the old and the modern dietetic treatment of disease, the laws of digestibility, convalescent food, &c., and will be illustrated by diagrams. This being the introductory lecture, the charge for admission is 1s. for members and non-members alike. For subsequent lectures non-members will in all cases be charged 6d. more than members of the club. Tickets from the Secretary at the club 1s. each; if to be sent by post, a stamped addressed envelope must accompany application.

### Notes and Queries.

The contents of the Editor's Letter-box have now reached such unwieldy proportions that it has become necessary to establish a hard and fast rule regarding Answers to Correspondents. In future, all questions requiring replies will continue to be answered in this column without any fee. If an answer is required by letter, a fee of half-a-crown must be enclosed with the note containing the enquiry. We are always pleased to help our numerous correspondents to the fullest extent, and we can trust them to sympathise in the overwhelming amount of writing which makes the new rules a necessity. Every communication must be accompanied by the writer's name and address, otherwise it will receive no attention.

*Dispensing.*

(144) Can you tell me where I can be trained in dispensing?—*R. M. P.*

Write to Miss Bradbury, resident dispenser at the Ryde Dispensary, Isle of Wight. This lady takes pupils, and will be able to give you the best advice.

(145) Could one qualify as a dispenser whilst continuing daily nursing at the same time? What books should be studied, and please give information about examinations?—*Constant Reader.*

Read answers to above queries. Certainly you could not combine training in dispensing and active nursing work at the same time, if, as you seem to imply, you wish to study for the examination of the Apothecaries Society or the Pharmaceutical Society. Read the chapter on dispensing in Burdett's "Hospitals and Charities" for 1894, where you will find a list of books for study. You should write to the Secretary of the Pharmaceutical Society, Bloomsbury Square, W.C., and the Secretary of the Apothecaries Hall, Blackfriars, E.C., for syllabus and full details of examinations and requirements for candidates. You could probably obtain practical instruction at a local hospital, or from a chemist.

*The Plague in India.*

(146) Is it true that nurses are wanted to volunteer for work amongst the victims of the plague in India, and if so, to whom should one apply?—*C. H. S.*

No, we have not heard that nurses are wanted, and do not think there is any call for volunteers. The plague is confined to the poorer native inhabitants, who for the most part remain in their homes rather than be taken to hospital, and under these circumstances we do not think English nurses should be allowed to undertake the work of nursing them.

*Missionary Work.*

(147) Kindly tell me where I can obtain information respecting nursing abroad, especially in connection with missionary work?—*F. A.*

Apply to the Church of England Zenana Missionary Society, 9, Salisbury Square, Fleet Street, E.C.; or to the Zenana Bible and Medical Mission, 2, Adelphi Terrace, W.C.

*Home for Inebriates.*

(148) Can you tell me of a home where a gentleman of occasional inebriate habits could be received on very low terms by giving his services? He is well educated, but would work in any capacity.—*Nurse Gertrude.*

See list of such homes in Burdett's "Hospitals and Charities" (Scientific Press, 28 and 29, Southampton Street, Strand, London, W.C.), page 850. The following homes take male patients, and you had better write to the superintendents and give particulars of the case in question: Kingswood Park, Bristol; Dalrymple Home, Rickmansworth, Herts; Highshot House, St. Margaret's, Twickenham.

*Nurses' Badges.*

(149) When an institution wishes its nurses to wear a badge, whereby it may be known that they are really certificated nurses, and no one but themselves are allowed to wear the same, have they not to be registered?—*Enquirer.*

There is no obligation to register a badge. If it is desired to do so apply to the Comptroller, Patent Office, Trade Marks Branch, 25, Southampton Buildings, W.C. Unless such badges are registered, of course they can be copied by anyone who likes to do so.

*Holt-Ockley System.*

(150) Where can I obtain particulars of this system?—*Sister.*

Write to the hon. secretary, Mrs. H. L. Steere, The Cottage, Ockley.

*Home Wanted.*

(151) Can you tell me of a home where a poor boy, age about nine, who is both idiot and epileptic, might be taken in? The guardians might pay something for him?—*H. A. F.*

A very difficult case for which to find a refuge. Have you applied at Earlswood Asylum? If not, we should suggest your writing to the Secretary. If the case is not eligible, possibly he might be able to suggest some other institution. Have you applied at the County Asylum?

*Ambulance Lectures.*

(152) Can you tell me if there are any ambulance lectures now being given in London open to anyone to attend?—*M. D. G.*

Write to the Secretary, St. John Ambulance Association, St. John's Gate, Clerkenwell, E.C.

